# Defining the altered glycoproteomic space of the early secretory pathway by class I mannosidase pharmacological inhibition

**DOI:** 10.3389/fmolb.2022.1064868

**Published:** 2023-01-09

**Authors:** Cristian V. A. Munteanu, Gabriela N. Chirițoiu, Andrei-Jose Petrescu, Ștefana M. Petrescu

**Affiliations:** ^1^ Department of Bioinformatics and Structural Biochemistry, Institute of Biochemistry, Bucharest, Romania; ^2^ Department of Molecular Cell Biology, Institute of Biochemistry, Bucharest, Romania

**Keywords:** glycoproteomics, glycosylation, protein degradation, mass-spectrometry, core-fucosylation

## Abstract

N-glycosylation is a key process for various biological functions like protein folding, maturation and sorting for the conventional secretory compartment, cell-cell communication and immune response. This is usually accomplished by a complex system of mannosidases in which those from class I have an outstanding role, commonly involved in the early protein sorting associated to the Endoplasmic Reticulum (ER) in the N-glycan dependent quality control (ERQC) and ER-associated degradation (ERAD). Although these are vital processes in maintaining cellular homeostasis, large-scale analysis studies for this pool of molecules, further denoted as proteins from the early secretory pathway (ESP), were limited addressed. Here, using a custom workflow employing a combination of glycomics and deglycoproteomics analyses, using lectin affinity and selective Endoglycosidase H (Endo H) digestion, we scrutinize the steady-state oligomannosidic glycoprotein load and delineate ESP fraction in melanoma cells. All of these were assessed by applying our workflow for glycosite relative quantification of both the peptide chain and carbohydrate structure in cells with inhibited activity of class I mannosidases after kifunensine treatment. We found that most of the ESP are transient clients involved in cell communication *via* extracellular matrix, particularly integrin-mediated communication which adopt Man9 N-glycans in kifunensine-treated cells. Moreover, our results reveal that core-fucosylation is decreased subsequent inhibition of class I mannosidases and this could be explained by a general lower protein level of FUT8, the enzyme responsible for fucosylation. By comparing our data with results obtained following downregulation of a key mannosidase in misfolded protein degradation, we mapped both novel and previously suggested endogenous substrate candidates like PCDH2, HLA-B, LAMB2 or members of the integrin family of proteins such as ITGA1 and ITGA4, thus validating the findings obtained using our workflow regarding accumulation and characterization of ESP transitory members following mannosidase class I inhibition. This workflow and the associated dataset not only allowed us to investigate the oligomannosidic glycoprotein fraction but also to delineate differences mediated at glycosite-level upon kifunensine treatment and outline the potential associated cellular responses.

## 1 Introduction

Approximately one-third of the total synthesized cellular proteins enter the secretory pathway (Barlowe and Miller, 2013). This imposes tight restrictions on the early quality control mechanisms meant to selectively remove misfolded or unfolded proteins which, if accumulated, can constitute the onset for several pathologies like neurodegenerative disorders, cancer, cystic fibrosis or Gaucher’s disease (Chaudhuri and Paul, 2006). One of the most important factors in protein folding, particularly for proteins with chaperone-assisted folding within the Endoplasmic Reticulum (ER) is related to glycosylation, mostly N-glycosylation. This takes place co- or post-translationally in the Endoplasmic Reticulum (ER) and is usually used to alter local polypeptide physicochemical properties to help attain the protein’s native conformation, but also to signal the protein’s ‘folding-state’ (Jayaprakash and Surolia, 2017). Proteins are N-glycosylated by the direct transfer of an oligomannose structure composed of Glc3Man9GlcNAc2 (G3M9) to the amide side-chain group of selected asparagine (Asn) residues (Aebi, 2013). After several rounds of chaperone-mediated folding cycles, which involves glycan trimming by specific glucosidases (Zapun et al., 1997) misfolded glycoproteins are finally selected for glycoprotein-ER-associated degradation (gpERAD). This involves the cooperation of an intricately network of mannosidases that trim the carbohydrate structure to much simpler ones with fewer mannoses (3-4 mannose residues) which are further recognized by specific lectin players in gpERAD transferring the faulty substrate to the cytosol by the retrotranslocation machinery, for proteasomal degradation (Liu et al., 1999). These events, affecting the incipient phases of the protein’s life, take place in the early compartments of the secretory pathway, predominantly ER, in the specific intracellular route previously defined as Early Secretory Pathway (ESP) (Anelli and Sitia, 2008; Sicari et al., 2020). Although these are vital processes for cellular homeostasis, currently there are only few studies focusing on the analysis of this molecular fraction (Zhang et al., 2011), none analyzing this pool in steady-state and altered conditions. Here, we have undergone a close examination of this fraction by using a dedicated workflow, which integrates lectin affinity, Endo H digestion and high-resolution mass spectrometry (HRMS) in the glycoprotein analysis of the Early Secretory Pathway (gESP). We used as a model system a melanoma cell line stably expressing a truncated form of the autoantigen tyrosinase which is decorated with seven N-glycosylation sites, all oligomannose-type glycan structures, sensitive to class I mannosidase processing. We took advantage of these aspects to validate our workflow and guide our analysis towards selecting ESP candidates. We found that most of the oligomannosidic glycoproteins are involved in extracellular matrix (ECM) organization and in integrin-dependent ECM-cell surface interactions, which indicate glycoproteins mediating a crosstalk between cell-cell and cell-microenvironment communication. We used our workflow to further delineate endogenous class I mannosidase candidates by relative quantification of the identified glycopeptides in control (CTR) and kifunensine (KIF) treated cells. This is a major selective inhibitor of class I mannosidases, enzymes involved in the early quality control steps from ESP. Our results revealed PCDHGC3, HLA-B, ITGA4 and LRP1 as major components of ESP protein fraction that accumulates following kifunensine treatment, these proteins having also key-functions in different pathologies such as pancreatic cancer (HLA-B) (Sliker et al., 2020), melanoma and ovarian cancer (ITGA4) (Hoek et al., 2004; Zhu et al., 2020) or Alzheimer disease (LRP1) (Shinohara et al., 2017). Remarkably, we also observed core-fucosylation downregulation of oligomannose and hybrid glycosites in KIF treated cells, accompanied by lower α-1,6-fucosyltransferase (FUT8) protein level. Our current dataset describes the major Endo H sensitive glycoprotein fraction and reveals an interesting cross-talk between class I mannosidase processing of glycoproteins and core-fucosylation.

## 2 Materials and methods

### 2.1 Cell culture and kifunensine treatment

A375 cells stably expressing the soluble version of human tyrosinase (A375-ST Tyr) were grown in DMEM (Gibco-31966–021) supplemented with 10% FBS (Gibco) and 400 μg/ml Geneticin (Invivogen) as previously described (Chiritoiu et al., 2016). Cells at confluence of 65%–70% were treated with 30 μM kifunensine (Santa Cruz Biotechnology) overnight (ON) or 20 μM MG132 (Santa Cruz Biotechnology) for 4 h and harvested accordingly.

### 2.2 SDS-PAGE and Western Blot analysis

Pelleted cells were lysed in Triton X100 containing buffer as previously described (Munteanu et al., 2019; Chiritoiu et al., 2020) or in RIPA buffer (25 mM Tris-HCl pH 7.6, 150 mM NaCl, 1% NP-40, 1% sodium deoxycholate, 0.1% SDS), supplemented with a cocktail of protease inhibitors (Roche). Equal amount of protein from each sample was treated or not with Endo H, following the enzyme manufacturer protocol (NEB) and separated in polyacrilamide gels. The proteins were transferred on nitrocellulose membranes and probed with mouse anti α-1,6-fucosyltransferase (FUCT8) (sc-271244), mouse anti-protocadherin 2 (PCDH2/PCDHGC3) (sc-376885), mouse anti-tyrosinase (T311) (sc-20035) antibodies from Santa Cruz Biotechnology and rabbit anti-tubulin as loading control (Abcam-18251). The membranes were washed and further incubated with secondary HRP coupled specific antibodies (Jackson ImmunoResearch). Proteins were detected with Immobilon Crescendo Western HRP substrate (Merk Millipore) and visualized at a Chemi Doc Imaging System (Bio-Rad). Band intensity from biological replicates were quantified using ImageJ software and represented as bar plots with error bars as SEM.

### 2.3 Isolation of partial deglycosylated glycopeptides

For protein extraction cells were lysed using the extraction buffer (EB: 100 mM Tris–HCl, pH 8.50, 6 M guanidine hydrochloride, 5 mM Tris (2-carboxyethyl)phosphine and 10 mM chloroacetamide) and incubated at 95°C for 5 –10 min. After sonication, the samples were centrifuged at 12,000 xg for 15 –20 min and subject to the Pierce Coomassie Plus analysis for total protein estimation. Equal amounts of CTR and KIF treated samples were loaded on a 10 K microcon device and further washed with digestion buffer (50 mM ammonium bicarbonate—ABC) before protease digestion. Samples were ON digested either with trypsin (Sequencing grade, Promega) in a 1:50 (w:w) ratio enzyme:protein or with GluC (Roche) at a ratio of 1:15, w:w enzyme:protein at 37°C. The peptides were collected directly in lectin binding buffer (LBB: 20 mM Tris–HCl, pH 7.50, 500 mM NaCl, 1 mM MnCl_2_, and 1 mM CaCl_2_) and transferred to a new microcon device along with 2x LBB Concanavalin A at a ratio of 1:2.1 (w:w, peptide:lectin) and further incubated for 1 h at room temperature (RT). The non-glycosylated material was removed by centrifugation at 10,000 xg, followed by four sequential washes of the microcon devices with LBB. For HexNAc peptide isolation, the microcon devices were further incubated ON at 37°C with 65 μl of 2 U/μl Endo H (1:2, peptides:enzyme) in G3 buffer (50 mM sodium acetate, pH 6.00). The resulting glycopeptides were recovered by centrifugation at RT for 20 min at 10,000 xg, followed by two additional washes of the microcon devices with G3 buffer. All the fractions recovered were combined and subject to C18-based stage-tip desalting.

### 2.4 Glycan isolation procedure

Endo H released glycans were recovered from EB lysates of CTR and KIF-treated cells using 10 K microcon devices. Samples were centrifuged at 10,000 xg in the device for protein retention. Subsequently the filtering units were washed with G3 buffer and further incubated ON at 37°C with 2 U/μl Endo H (1:2, protein:enzyme) as described in the enzyme datasheet. The released glycans were recovered in the same buffer following 10,000 xg centrifugation and by subsequent washes with water of the filter units. The combined fractions were further analyzed by HRMS.

### 2.5 Stage-tip desalting of the resulting glycopeptides

For sample desalting stage-tips were manually prepared in house with two to three Teflon-immobilized C18 disks. Before sample desalting each stage-tip was activated with methanol and then equilibrated first in 0.5% AcOOH (acetic acid) and 80% ACN (acetonitrile) and then in 0.05% AcOOH. Samples were acidified with AcOOH and then applied to the stage-tips disks, washed with 100 μl 0.5% AcOOH and then subject to elution with 2 × 75 μl of 0.5% AcOOH and 80% ACN. The combined fractions were further concentrated in a speed-vac and reconstituted in solvent A (0.06% formic acid - FA and 2% ACN) before further analysis.

### 2.6 Glycan analysis by high-resolution nanoES-MS/MS

The recovered oligomannose N-glycans samples were transferred to nanoESI emitters (Cat No. ES380, Thermo Fisher Scientific) and analyzed on an LTQ-Orbitrap Velos Pro instrument (Thermo Fisher Scientific) using the Nanospray Flex Ion Source kit (Wilm and Mann, 1996) (Thermo Fisher Scientific). Data acquisition was performed in positive mode by recording the ion current corresponding to the mass range expected for oligomannosidic glycans, in the Orbitrap portion of the instrument at a resolution of 100,000 (m/z 400). To obtain a stable spray the potential applied to the emitter was kept at ∼0.9–1.0 kV and the transfer capillary temperature was set to 275°C. For MS/MS analysis the observed ions corresponding to the released N-glycans were manually selected with 1–4 m/z isolation window and fragmented in the HCD collision cell with nitrogen (4.7 purity) at 40–55 collision energy (such that the characteristic fragments could be detected), followed by high-resolution detection in the Orbitrap. To obtain the best mass accuracy, before spectra acquisition, the m/z range 150–2000 m/z was calibrated with the external ESI positive calibration solution as recommended by the manufacturer.

### 2.7 HexNAc glycopeptide analysis using nanoLC-MS/MS

Before injection, samples were reconstituted in solvent A (0.06% FA and 2% ACN) and further analyzed using an Easy-nanoLC II (Thermo Fisher Scientific) coupled online to an LTQ-Orbitrap Velos Pro instrument (Thermo Fisher Scientific). The glycopeptides were loaded at a high flow (∼9–12 μl/min) on a RP C18 AcclaimTM PepMapTM 100 trap nanocolumn (ThermoFisher Scientific) and subsequently separated at low flow (∼300 nl/min) on a RP C18 AcclaimTM PepMapTM 100 analytical nanocolumn (ThermoFisher Scientific) using a 240 min 2%–30% solvent B (0.06% FA and 80% ACN) gradient. For analysis, the instrument was set to data dependent acquisition (DDA) using the LTQ Tune Plus (v 2.7) and Xcalibur v3.0 platforms. A top 10 method was used in which an initial Orbitrap survey scan at 30,000 resolution (m/z 400) was followed by the fragmentation in the HCD collision cell of top 10 the most intense ions from the m/z 300–1,550 region. The glycopeptide fragment ions were further transferred and detected in the Orbitrap section of the instrument at a resolution of 7,500 (m/z 400). To increase the mass accuracy the internal calibration for the lock mass of 445.120,025 was used for a spray voltage of ∼1.8–2.2 kV and a transfer capillary temperature of 275°–300°C. To reduce the redundancy of ions selected for fragmentation the dynamic exclusion option was activated for 30 –60 s duration with a repeat count of one and a maximum list size of 500. Exclusion lists were used for samples with multiple injections.

### 2.8 Raw MS and MS/MS data analysis

For HexNAc glycopeptide data analysis the raw files corresponding to each sample technical and biological replicate were searched against the human version of the UniProtKB database (98,758 sequences as of 04/2021) using the Andromeda algorithm integrated into the MaxQuant environment v1.6 with Trypsin/P (with maximum two missed cleavages) as the selected protease or/additionally GluC (DE with maximum four missed cleavages) including the following modifications: Carbamidomethyl on Cys residues as a fixed modification and Met oxidation, HexNAc or dHexHexNAc on Asn residues and Acetyl on the protein N-terminus as variable modifications. For precursor ions the mass accuracy for the first search was set at 20 ppm and 7 ppm for the second search, while for the fragments ions a maximum of 0.02 Da was used. The match between runs options was activated with an alignment time window of 20 min and a matching time window of 1 min. The identifications were filtered for 1% FDR (False Discovery Rate) at PSM (Peptide Spectrum Match) using the built-in MaxQuant procedure. For glycan MS and MS/MS analysis the raw files were manually analyzed by searching the corresponding masses of the oligomannosidic glycans within the expected mass tolerance (10 –20 ppm). Glycan mass calculations were performed using NIST Glyco Mass Calculator (Huang et al., 1999), for both, precursor and fragment ions assuming no particular glycan modifications (Supplementary Table S4). Only Endo H-like released structures were considered for carbohydrate assignments. For quantification of the glycan relative abundance 50–60 MS spectra were used for ion count averaging between 750 and 2000 m/z with an approximately equal total ion count.

### 2.9 Dataset bioinformatics analysis and validation

For glycopeptide analysis, entries corresponding to contaminants and reverse identifications were first removed and only records with a PEP<0.01, Andromeda score of at least 40 and a delta score of minimum eight were further kept for analysis. Furthermore, only MS/MS spectra containing the diagnostic oxonium ion were considered and glycosites with at least 0.75 localization probability and a minimum score difference of at least five were included for the final output. MS/MS spectra with ambiguous identifications were further manually verified. For relative quantification the intensity values were exported, log transformed and only identifications with at least three quantified values either in the CTR or KIF-treated sample were included. Missing values were replaced with ones from a normal distribution with a lower mean, assuming these were close to the instrument detection limit. For Gene Ontology (GO) and pathway analysis the DAVID annotation system was used (Sherman et al., 2022) and only entries statistically significant (Ease score <0.05) according to Bonferroni correction were considered.

## 3 Results

### 3.1 Decoding the oligomannosidic N-glycoprofile landscape using glycoESP (gESP) workflow

**FIGURE 1:**
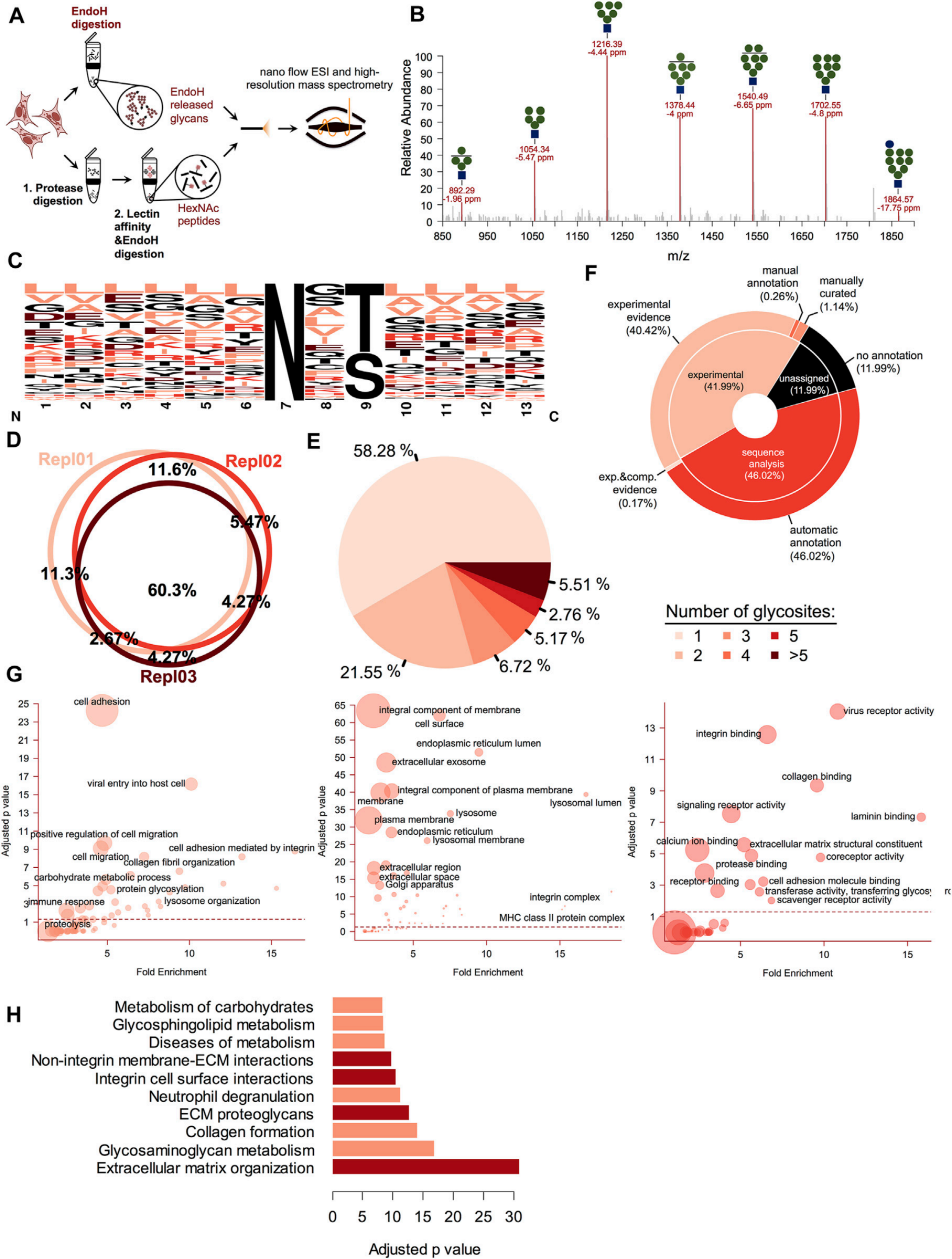
Glycoproteomic analysis of the early secretory pathway (ESP) members. **(A)**. Schematic illustrating the principle of the gESP workflow for the analysis of oligomannosidic and ESP glycoproteins. To analyze both, the carbohydrate and the protein fraction from the same sample, the extracted proteins from the biological material are split. Glycans are released using Endo H digestion and another part is subject to protease digestion 1) and the glycopeptides are enriched using lectin-affinity 2). To obtain the same pool of non-mature glycoproteins, these are further released using Endo H digestion. Both fractions containing released N-glycans and HexNAc peptides are further analyzed using high-resolution mass spectrometry (HRMS). **(B).** Oligomannose glycan profile obtained from a cellular lysate of A375 melanoma cells subject to Endo H digestion. These were identified as single charged sodium adducts in nanoESI for the full range from G1M9 to G0M4. Shown are the observed m/z, and the calculated mass accuracy. **(C).** Sequence logo of the corresponding identified glycosites. Most of the identified glycosites correspond to the N-X-T/S motif. **(D).** Biological replicate analysis of the identified glycosites. More than 60% of the glycosites are reproducible identified. **(E).** Glycosite distribution analysis across the identified glycoproteins. **(F).** UniProtKB annotation of the identified glycosites for each major category: those with experimental data, those annotated only based on sequence or not mentioned glycosites. **(G).** Gene Ontology annotation for each major class (Biological Process—left panel, Cellular Component—middle panel and Molecular Function—right panel) of the identified glycoproteins. Shown are the fold enrichment and Bonferroni adjusted *p*-value. The circle area reflects the number of members found in each category. **(H).** Pathway analysis using Reactome database information. Top enriched pathways based on the Bonferroni adjusted log *p*-value. Most of these refer to cell surface-ECM interactions mediated by various pathways (integrin-based or alternative routes).

Most of the ESP glycoproteins acquire various oligomannose derived carbohydrates by incorporation of the (Glc)_3_ (Man)_9_ (GlcNAc)_2_, denoted as (G3M9) structure on the nitrogen atom from the amide group of selected Asn residues from the polypeptide chain (Aebi, 2013). These oligomannose and hybrid structures are released by Endo-β-N-acetylglucosaminidase H (Endo H) enzyme, which is frequently used to study glycoprotein maturation along the secretory pathway (Tarentino et al., 1974; Trimble and Tarentino, 1991). In gESP the proteins extracted from a biological sample are divided in two, one part is used to enzymatically release N-glycans and the second one is used to enrich in oligomannose containing glycoproteins using lectin affinity (Figure 1A). Finally, the latter are also released enzymatically and both of the obtained fractions are separately analyzed using high-resolution mass spectrometry (HRMS). This workflow results in several advantages such as: first it restricts the analysis only to oligomannosidic glycoproteins, using Endo H specificity to fractionate the pool of captured glycoproteins only to the ones of interest and secondly, it eases the burden of intact glycopeptide identification from MS/MS spectra, which usually contains mixed fragments of both peptide and carbohydrate structures and are likely more difficult for large scale automated assignment (Hu et al., 2017). Moreover, it can provide information not only on glycosite position, but also on the maturation state of the captured glycosites, a critical element for biological activities like folding, degradation, secretion and in general glycoprotein subcellular traffic. We first assessed our workflow for the detection of the Endo H sensitive N-glycans decorating glycoproteins. For this, proteins extracted from A375 melanoma cells stably expressing soluble tyrosinase were subjected to overnight Endo H digestion in a 10 K microcon device and the released glycans were further captured by centrifugation and analyzed by HRMS. As it can be observed in Figure 1B, we obtained the oligomannosidic glycan profile using nanoflow electrospray from A375 melanoma cells and detected structures extending from G1M9 to G0M4 with mass accuracies lower than 7 ppm, in general. These, were identified as sodium salts of native glycans and high-resolution MS/MS, confirmed the assigned structures (Supplementary Figure S1A).

We next established the ability of our workflow to capture their corresponding glycosites. One important advantage of this setup is that following lectin enrichment and Endo H cleavage all the enriched glycopeptides still contain one GlcNAc (HexNAc) residue at the glycosylation site. This provides additional evidence for glycosylation compared with the classical PNGase F-based deglycoproteomics methods which usually use deamidation as indirect evidence for glycosylation (Palmisano et al., 2012). After careful filtration of the data to rule-out potential false positive (as described under Materials and Methods section) we identified close to 1,200 glycosites mapped on more than 570 UniProtKB entries (Supplementary Table S1). Similar to the associated glycans, these were detected, in general with mass deviations of less than 5 ppm (Supplementary Figure S1B), thus increasing the confidence of the identifications. Considering we targeted only a small fraction from the total glycoprotein pool and that we observed the reproducible identification of more than 60% of the glycosites between biological replicates (Figure 1D) we concluded that we obtained a good coverage for the current dataset. Approximately 98% (1,133) of the identified glycosites corresponded to the N-linked glycosylation motif N-X-S/T (where X is any amino acid except proline, Figure 1C). We also observed a slight preference for threonine instead of serine residues in the second position of the sequon (about 1.5 fold frequency ratio). Out of the remaining glycosites, 13 sites were found to contain Cys or Val instead of Ser or Thr, further confirming previous observations regarding the NXC/NXV glycosylation motifs (Supplementary Figure S1C, D) (Bause and Legler, 1981; Zielinska et al., 2010), as most probably these are typical substrates of STT3B (Cherepanova and Gilmore, 2016; Cherepanova et al., 2019). Analysis of glycosite distribution across the identified proteins revealed that almost 58% had a single glycosylation site and ∼22% two glycosylation sites, this covering roughly 80% of the identified glycoproteins (Figure 1E). This is similar with the distribution observed for the full N-glycosylation repertoire captured with PNGase F (Deeb et al., 2014), thus denoting that these follows close similarity. However, we also identified some heavily glycosylated proteins such as Prolow-density lipoprotein receptor-related protein 1 (LRP1) with 18 glycosylation sites, Laminin subunit alpha-5 with more than 10 glycosylation sites or Integrin alpha 3 (ITGA3) with 10 sites and Laminin subunit beta-1 with nine glycosites. Only 42% of the glycosites had been previously annotated in the UniProtKB database based on experimental evidence, whereas 46% were annotated based on the sequence analysis (Figure 1F). Thus, about 58% of glycosites identified in this study were either confirmed experimentally or identified *de novo*, since some were not even annotated as potential glycosylated.

Gene Ontology analysis revealed that most of the identified glycosites correspond to proteins involved in cell adhesion, cell migration, lysosome organization or carbohydrate-related biological processes (Figure 1G, left panel). This was also sustained by the cellular localization analysis, as most of the identified proteins were associated with cell surface, plasma membrane or various intracellular compartments like endoplasmic reticulum or lysosomes (Figure 1G, middle-panel). This confirms that most of the glycoprotein pool is represented by late secretory pathways proteins, in transit to other subcellular compartments such as plasma membrane or lysosomes. Regarding their biological function, the identified glycosites originated from proteins involved in both integrin-mediated cell-ECM (Extracellular Matrix) interactions but also in alternative pathways involving interactions with various receptors, collagen or glycosaminoglycan, confirmed by both the distribution of molecular functions terms associated with these and by the reactome pathway analysis, which revealed numerous pathways linked to ECM organization and communication (Figure 1G right panel and Figure 1H). In summary, all these aspects confirm that our workflow is as a reliable tool for oligomannosidic glycoprotein fraction characterization, usually associated with ESP.

### 3.2 gESP workflow captures the repertoire of kifunensine altered glycosites

**FIGURE 2:**
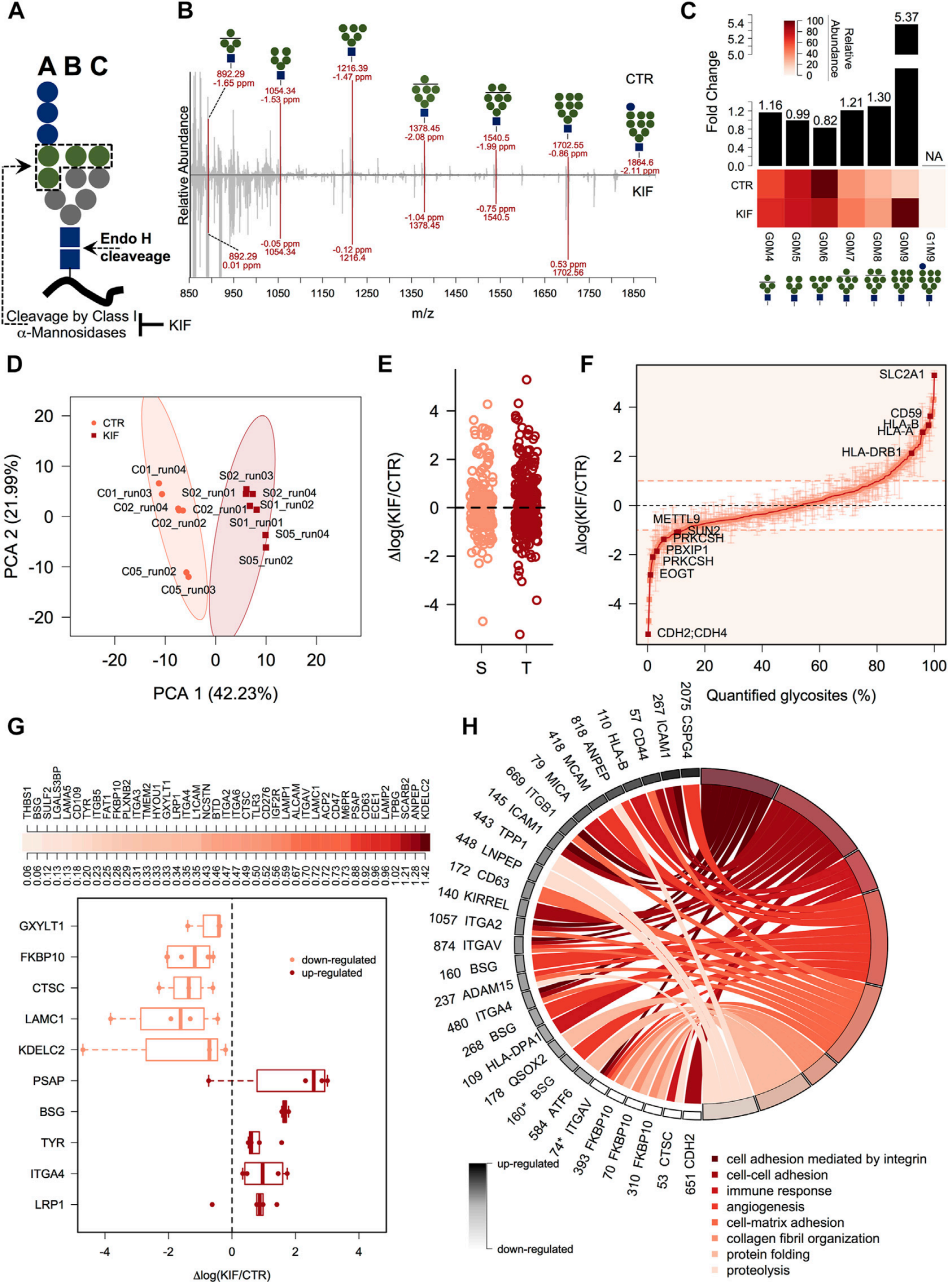
gESP analysis of kifunensine treated cells. **(A).** General structure of the N-linked glycan transferred on N-glycosylated proteins during intracellular glycosylation. Shown are the Endo H cleavage point and the three main branches. Green circles denotes α1,2-linked mannose residues which are recognized by class I mannosidases. **(B).** Mirror plot of the Endo H released carbohydrate structures from CTR and KIF treated cells. All major classes (G0M9 - G0M4) of released N-glycans were found in both conditions as sodium adducts. Shown are the m/z and mass accuracy from both conditions. **(C).** Analysis of the relative abundance for the major N-glycans identified in (B). The lower heat map displays the relative abundance of the identified classes and upper bars denote calculated fold change between KIF and CTR cells. **(D).** PCA analysis of the corresponding glycosites identified in the two conditions. **(E).** Analysis of the quantified glycosites containing Ser or Thr. No apparent differences were observed for the two categories. **(F).** Distribution at the glycosite level of the log fold changes between KIF and CTR cells. Points are median of biological triplicates and error bars denote SEM. Several top up-regulated and down-regulated glycosites are shown with their corresponding gene name. **(G).** Upper-panel: Distribution of calculated glycoprotein SEM across the identified glycosites. Lower-panel: Boxplot indicating top up-/down-regulated glycoproteins in KIF treated cells. Points denote each quantified glycosite. Only glycoproteins with at least three quantifiable glycosites were considered. **(H).** Biological pathway Gene Ontology (GO) analysis of the quantified glycoproteins. Shown are the gene names and glycosylation position with their corresponding up-/down-regulation in black to white colors and their associated biological processes in shades from dark to light-red. It can be observed the segregation of different biological processes with the estimated fold-change. Glycosylation positions refer to the UniProt reviewed entries of the corresponding gene names. * designates a dHexHexNAc site.

We next focused on quantitative aspects of our workflow and we quantified both, the Endo H released glycans and the associated glycosites for cells grown under two different conditions. We treated cells with kifunensine (KIF), an immunomodulatory agent (Iwami et al., 1987), which inhibits class I mannosidases (Elbein et al., 1990). Unlike class II mannosidases the ones from class I are responsible for cleavage only of α1,2 linked mannose residues from the N-linked glycan core (Figure 2A) (Daniel et al., 1994), generating various trimmed products from G0M9 to G0M5. Most of their targets are glycoproteins from ESP, which are Endo H sensitive. We thus performed our workflow with control (CTR) and KIF treated cells and relatively quantified both the fraction of N-glycans and their associated glycosites. Upon KIF treatment we observed an increase in unprocessed glycoforms particularly G0M9 (Figures 2B,C) and a slight decrease in processed forms such as G0M6. This demonstrates that the ESP glycoprotein fraction is targeted by KIF treatment and most of the class I mannosidases glycoprotein substrates accumulate as unprocessed G0M9 glycoforms. Further analysis of the corresponding glycosite relative intensities revealed that using our workflow we were able to capture relative differences between CTR and KIF-treated cells, at the glycosite level, reflected by both the PCA analysis where we observed a clear spatial discrimination (Figure 2D) and by the correlation heatmap which revealed strong associations between replicates from the same condition (Supplementary Figure S2A). Nevertheless, we did not observed particular shifts of the intensity differences distribution between the two conditions (Supplementary Figure S2B), or when comparing distributions across Ser or Thr containing glycosites (Figure 2E). Altogether, these confirm that using our workflow it is currently possible to capture differences at the glycoproteome level with amino acid resolution thus characterizing both the carbohydrate and the peptide fraction. More than one-third (157) of the quantified glycosites displayed altered intensities compared with the CTR cell line (differences of at least a single unit in log scale) with a slightly higher percent of up-regulated glycosites than the down-regulated ones (∼19% compared with 13%, Figure 2F and Supplementary Table S2). It is important to mention that the values refer strictly to the relative abundance between the two conditions and not to the absolute occupancy in either of them. Close inspection of the data revealed downregulation of glycosites, under KIF treatment, associated to various proteins such as lysosomal proteins (Lysosomal alpha-glucosidase - GAA), membrane proteins (Carboxypeptidase D - CPD), ER-resident (EGF domain-specific O-linked N-acetylglucosamine transferase—EOGT, the regulatory subunit of Glucosidase 2 – PRKCSH) or secreted (Tumor necrosis factor receptor superfamily member 5 - CD40) as observed in Figure 2F and Supplementary Table S2. Typical examples of upregulated glycosites involved mainly proteins from plasma membrane (Cell surface glycoprotein MUC18 - MCAM, Solute carrier family 2, facilitated glucose transporter member 1 – SLC2A1) but also various shuttling glycoproteins between ER and plasma membrane or Golgi and plasma membrane such as different HLA isoforms or respectively CMP-N-acetylneuraminate-beta-galactosamide-α-2,3-sialyltransferase 4 – ST3GAL4, a beta-galactoside α2-3 sialyltransferase involved in terminal sialylation of glycoproteins and glycolipids (Kitagawa and Paulson, 1994; Basu et al., 1996), Figure 2F and Supplementary Table S2. A particular question that arises is to what extent these differences are a consequence of the altered glycoprotein level upon kifunensine treatment or reflect a single variation at a particular glycosite, thus encoding differences in glycosite macro- and microheterogeneity. As previously mentioned (Rademacher et al., 1988; Sumer-Bayraktar et al., 2012; Čaval et al., 2021), we define macroheterogeneity as presence or absence of the N-glycan at a particular site (that is glycosite occupation) and microheterogeneity as the occurrence of various carbohydrate structures on the same glycosite. This assumes that kifunensine treatment would not impact at the same level every glycosite of a particular glycoprotein thus resulting in variation across the measured relative differences of the same protein. For this reason, we selected glycoproteins with at least three quantifiable glycosites and calculated the log fold change variability across the protein glycosites expressed as standard error of the mean (SEM). We choose SEM and not SD, since SEM considers the number of measurements taken into account, thus favoring glycoproteins with numerous glycosylation sites. This allowed us to rank the entries based on SEM, regardless of the relative difference level. We found downregulated glycoproteins such as Dipeptidyl peptidase 1 – CTSC (a lysosomal thiol protease), FKBP10 (a peptidyl-prolyl cis-trans isomerase involved in protein folding), or upregulated ones such as LRP1 (a receptor for lipoproteins) and other member of integrin family—ITGA4 with SEM values less than 0.5, which is approximately 30% from the maximum calculated SEM suggesting alteration at the overall glycoprotein level in cells treated with kifunensine (Figure 2G upper panel). Similarly in this category we also found some positive controls such as the canonical ERAD substrates CD147 (Basigin—BSG) (Tyler et al., 2012), or the soluble version of tyrosinase (Munteanu et al., 2021). A particular class of glycoproteins is represented by those exhibiting complex regulation following kifunensine treatment, in which distinct glycosites displayed opposite relative log fold changes on the same protein. In this group we identified glycoproteins such as the Endothelin-converting enzyme 1 (ECE1), Prosaposin (PSAP) or Lysosome-associated membrane glycoprotein 2 (LAMP2), which displayed a SEM value (0.96) close to more than 50% of the maximum (1.42, see Figure 2G upper-panel and Supplementary Table S2). Considering our overall good reproducibility, we consider that most of these unexpected regulations do not reflect technical errors but rather glycosites-specific alterations that can be induced by class I mannosidase processing (microheterogeneity), isoform-specific alterations or, alternatively, could reflect a reduced glycosylation of a specific glycosite (macroheterogeneity).

### 3.3 Kifunensine disturbs the overall processing of cell surface glycosites from plasma membrane glycoproteins

One question that arises is to what extent kifunensine treatment impacts the glycosites or glycoproteins related to specific biological activities or compartments. Therefore, we next focused on the annotation analysis of the glycoproteins found with different relative abundance in kifunensine treated cells. We annotated the glycoprotein list with the subcellular localization and topology information from the UniProtKB database to further analyze the data. As can be observed in Supplementary Figure S2C, the results revealed that most of the up-regulated glycosites originated from plasma membrane proteins (∼74%) with glycans exposed to the extracellular surface (60%). Secondly, around 9% of the up-regulated glycosites were from lysosomal proteins, closely followed by Golgi and secreted ones. For the down-regulated glycosites, we observed a slightly lower representation of the plasma membrane proteins (∼40%), compensated by a net increase of ER glycoproteins (from 3% found in the up-regulated list to 29% for the down-regulated ones, Supplementary Figure S2C). Interesting, we found a lower percent of Golgi-annotated proteins in the down-regulated list, suggesting that kifunensine treatment would impact only ER-resident Endo H sensitive glycoproteins. We further annotated our list with Gene Ontology (GO) - Biological Process (BP) specific terms to delineate the biological function of the glycoproteins impacted by kifunensine treatment. We found that most of the upregulated glycosites were found on glycoproteins responsible for integrin-mediated cell adhesion (Figure 2H, dark-red colors), closely followed by up-regulated glycosites from proteins involved in regulation of immune response. The down-regulated ones were mainly involved in protein folding (Figure 2H, light red colors). This is in agreement with our observation regarding kifunensine-mediated downregulation of multiple ER-resident proteins (∼29%, Supplementary Figure S2C) involved in protein folding, trafficking and degradation such as Peptidyl-prolyl cis-trans isomerase FKBP10 (FKBP10), Lysosomal alpha-glucosidase (GAA) or to stress response, Clusterin—CLU (Supplementary Table S2). These results point to a broader picture in which most of the upregulated glycosites in kifunensine treated cells originate from transitory glycoproteins with the final destination to plasma membrane involving glycosites with topological cell surface exposed carbohydrates responsible for cell adhesion containing more or less integrin mediated connections. It also appears that the downregulated glycosites do not show a similar bias but are more evenly distributed between various intracellular compartments (ER, Golgi, Lysosome etc). Thus, using our workflow we mapped the class I mannosidases cellular targets, with single glycosite-specific resolution and revealed that most of these are transitory ESP-associated glycoproteins adopting Man9 structures, following kifunensine treatment.

### 3.4 Protein core-fucosylation of ESP glycoproteins is downregulated in kifunensine treated cells

**FIGURE 3:**
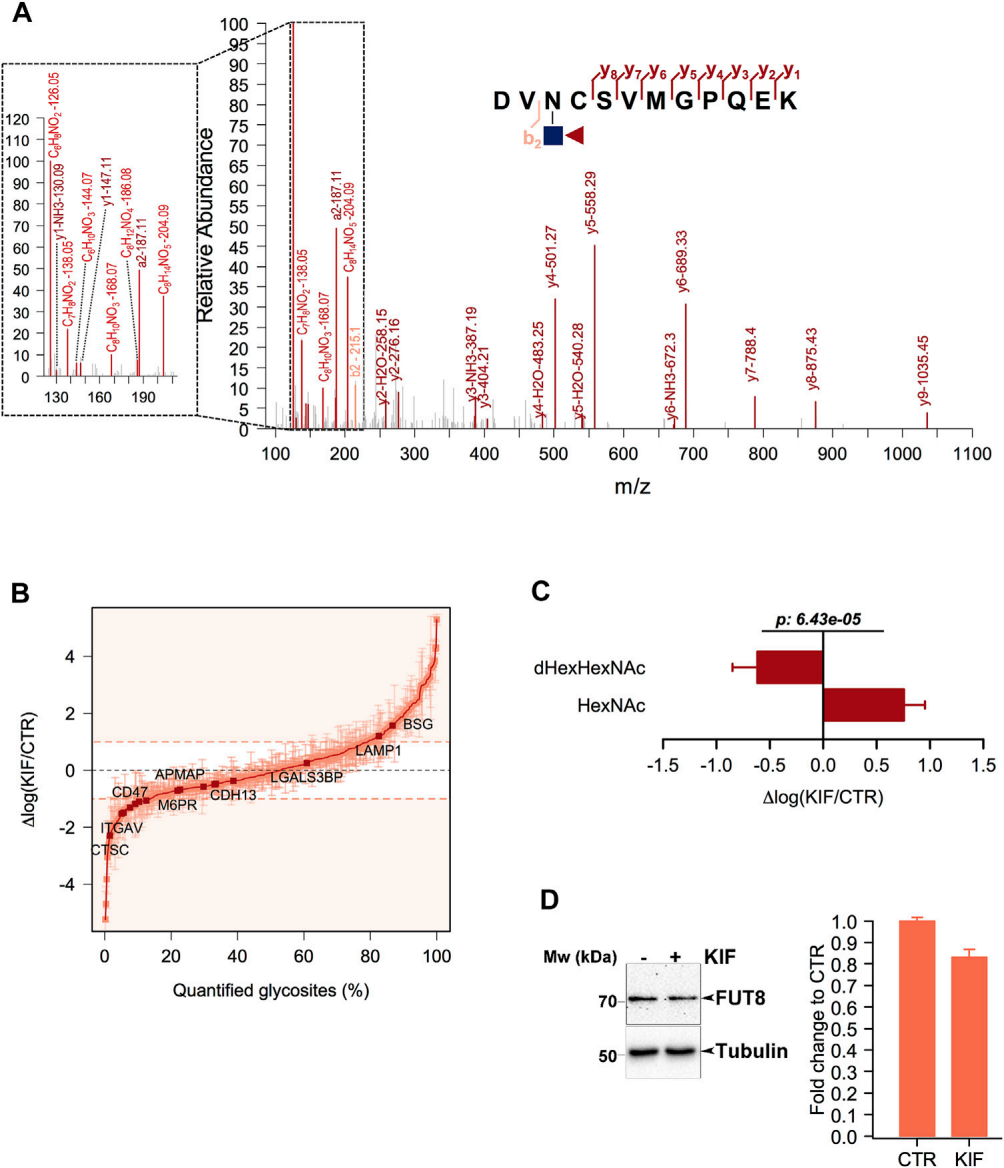
Analysis of core-fucosylated glycosites in KIF treated cells. **(A).** HCD MS/MS spectrum of the glycopeptide DVN(dHexHexNAc)CSVMGPQEK. Shown are the b and y ions. Inset: Low mass oxonium diagnostic ions identified in the MS/MS spectrum which confirms the modification. **(B).** Distribution of the log fold changes between KIF and CTR cells for core-fucosylated glycosites. Most of these show a lower relative abundance in KIF treated cells. **(C).** Analysis of the glycosites fucosylated and of the corresponding non-fucosylated glycosites from the same glycoproteins. Bars are mean of the core-fucosylated glycosites (n = 15) and their corresponding non-fucosylated glycosites (n = 28) from the same glycoproteins and error bars are SEM. For statistical significance a Welch two sample *t*-test was used. **(D).** Left panel: Representative Western Blot of FUT8 relative expression in CTR and KIF-treated cells. Right panel: Estimated fold change after densitometry analysis of duplicate experiments as those in left-panel.

Core fucosylation is a biological process mediated by α-1,6-fucosyltransferase (FUT8), which involves the transfer of a fucosyl (Fuc) residue from GDP-Fuc to the innermost GlcNAc, next to the protein asparagine side chain linkage (Uozumi et al., 1996). Since previous works revealed that Endo H is able to cleave such modified oligomannosidic structures (Cao et al., 2018) we also investigated this modification during the analysis of our data. We identified several dHexHexNAc modified residues as shown in Figures 3A, B. Since Endo H does not cleave complex N-glycans (Trimble and Tarentino, 1991), this suggests that these glycoproteins contained either oligomannose or hybrid glycostructures, thus providing important information regarding their glycan maturation state. Although this fraction of molecules was not large (∼1% of the identified glycosites), we further interrogated our results regarding the relative log fold changes between CTR and KIF treated cells. We observed that most of the fucosylated glycopeptides displayed lower relative abundances in kifunensine, compared with CTR cells (Figure 3B). This was observed regardless of protein subcellular localization. To further investigate if this observation involves a general glycoprotein down-regulation or if this effect is glycosite specific we compared the relative abundances of all the identified glycosites from the same proteins. When comparing dHexHexNAc with HexNAc-derived glycosites of the same glycoproteins we observed a large difference between the relative abundance of the two glycopeptide classes in kifunensine treated cells. Moreover, most of the corresponding HexNAc identified glycopeptides displayed an overall increase in the treated cells (Figure 3C). Thus, the negative log values observed for the fucosylated glycostructures rather refer to those particular glycosites alteration and not to all glycosites down-regulation of the same protein, mediated by kifunensine treatment. However, down-regulation of glycoprotein core fucosylation in kifunensine treated cells could result following either due to a general downregulation of FUT8 protein, or to a general decrease of FUT8 enzymatic activity resulting in decreasing core-fucosylation or, alternative, to both. To find which of the assumptions could result in such observations and to further strengthen our results we analyzed by Western Blot (WB) the level of FUT8 protein expression in CTR and KIF treated cells. As it can be observed in Figure 3D, densitometric analysis of FUT8 protein, revealed a slightly lower abundance in KIF treated cells compared with CTR cells. This suggests that the overall lower protein core-fucosylation could be at least partially, explained by the differences in FUT8 relative abundance in the two conditions. However this does not rule out the possibility of a lower enzymatic activity of FUT8, due to the a lower specificity towards G0M9 and G0M8 glycostructures.

### 3.5 Comparative analysis between kifunensine and siEDEM2 treated cells reveals mannosidase -specific endogenous candidates and validates gESP workflow

**FIGURE 4:**
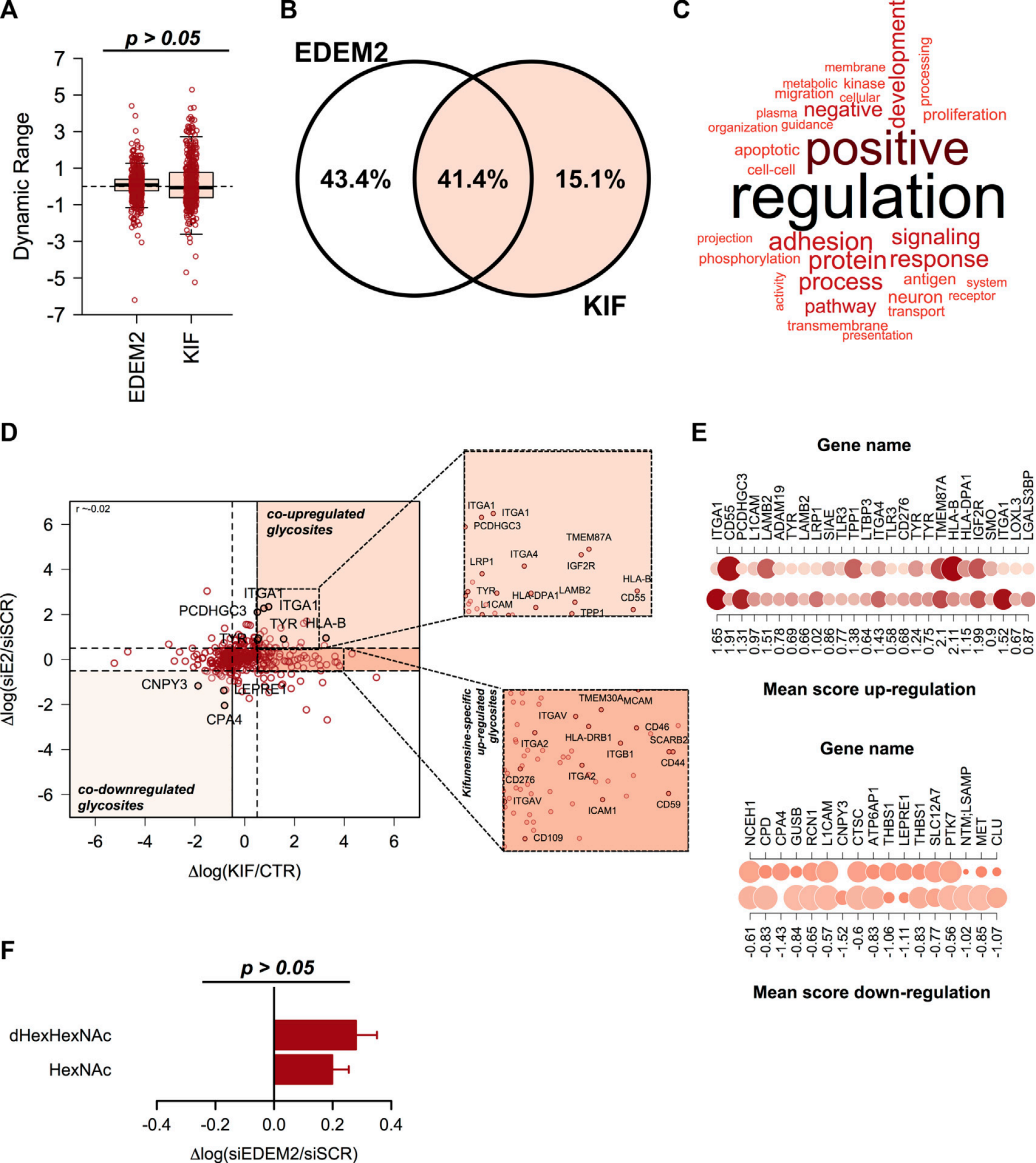
Comparison analysis of kifunensine and siEDEM2 treated cells. **(A)** Boxplot of the relative log fold changes between cells treated and their corresponding controls. No major changes were observed, except for a slightly larger distribution for the current dataset. **(B)** Venn Diagram of the glycosites identified in each of the analyzed datasets or in both experiments. **(C)** Gene Ontology (GO) analysis of the Biological Process (BP) terms associated with the glycoproteins identified in the merged dataset. **(D)** Distribution of the relative changes between the two analyzed conditions: log KIF/CTR and respectively log siEDEM2/siSCR. Each point denote a quantified glycosite in both experiments. Shown are some hits found up-/down-regulated in both experiments. Upper-inset: Zoom-in view with up-regulated glycosites in both datasets. Lower-inset: Zoom-in view for up-regulated glycosites only in this dataset. **(E)** Representation of the mean score for glycosites found up-regulated in both experiments (left-panel) and down-regulated in both siEDEM2 and KIF treated cells (right panel). **(F)** Analysis of the fucosylated glycosites and of the corresponding non-fucosylated glycosites from the same glycoproteins from EDEM2 dataset. Bars are mean of the core-fucosylated glycosites (*n* = 30) and their corresponding non-fucosylated glycosites (*n* = 68) from the same glycoproteins and error bars are SEM.

We recently published a dataset with candidate substrates (Chirițoiu et al., 2021) of EDEM2 (ER degradation-enhancing alpha-mannosidase-like protein 2) protein with a key-role in the selection and degradation of gpERAD clients. Previous reports have shown that EDEM2 is the first mannosidase involved in misfolded glycoprotein degradation, converting G0M9 to G0M8 structures on selected misfolded clients, by removing a single α1,2-linked mannose from branch B of the N-glycan core (Ninagawa et al., 2014; George et al., 2020). Since EDEM2 is considered a typical member of class I mannosidases and kifunensine inhibits all mannosidases in this class, we aimed to compare our published data with the current dataset. The previous data were obtained by comparing CTR and cells treated with small interfering RNA (siRNA) targeting endogenous EDEM2. We first extracted the ion current corresponding to all identified glycopeptides and compared the overall dynamic range. We did not observed significant deviations (Figure 4A), except for a slightly larger spread of the quantified values in KIF treated cells, which overall would be expected considering that in siEDEM2 dataset only a single mannosidase was targeted, compared with the current dataset in which multiple mannosidases were inhibited. When comparing the identity of the quantified glycosites, more than 40% were found in both datasets, which suggests a good coverage of the mannosidase class I targets (Figure 4B). Analysis of the GO BP terms for proteins with glycosites found in the merged dataset revealed enrichment of terms related to cell regulation response to various stimulus, protein metabolic process, immune response, matrix organization or cell adhesion (Figure 4C). We next compared the relative differences found in the two datasets for the shared fraction of glycosites and we found no particular correlation (Pearson correlation coefficient close to 0, Figure 4D). However, close inspection of the top upregulated glycosites found in both datasets revealed the identification of glycosylation sites from Protocadherin γ-C3 (PCDHGC3 or PCDH2), Integrin alpha-1 (ITGA1), HLA class I histocompatibility antigen, B alpha chain (HLA-B) and tyrosinase (TYR), same glycoproteins found as top reported candidate substrates for EDEM2 (Munteanu et al., 2021). This not only just confirms our previous results, but also validates our current findings, regarding enrichment of ESP-associated glycoproteins in kifunensine treated cells. As a consequence, we further interrogated the combined data for upregulated glycosites, which could denote additional EDEM2 clients. For this we scored glycosites found in both conditions with a similar trend (either up-regulated >0.5 log fold change units, or down-regulated < -0.5 fold change units in both datasets) by calculating the mean up/down-regulation score between the relative differences found in both datasets (Figures 4D, E). Beside the candidate hits mentioned earlier, higher scores were found for glycosites from proteins such as Integrin alpha-4 (ITGA4, mean up-regulation score of 1.43), Cation-independent mannose-6-phosphate receptor (IGF2R with score 1.99) or Laminin subunit beta-2 (LAMB2, with score 1.51), which were found as co-upregulated in both datasets, as shown in Figure 4E, upper panel. ITGA4 functions as a receptor for fibronectin, a protein involved in cell adhesion, with similar functions as LAMB2. As already noted, most of these are membrane proteins with cell surface exposed glycans. Similarly, among glycosites down-regulated in both datasets we found Carboxypeptidase A4 (CPA4 with a negative score of -1.43) or Protein canopy homolog 3 (CNPY3 score -1.52, Figures 4D, E, lower panel). Contrary to the up-regulated ones, these are either secreted (CPA4) or genuine members of the secretory pathway (CNPY3), thus reflecting contrasting effects in different subcellular compartments following EDEM2 activity inhibition. It is important to mention that the comparison refers strictly to the same glycosite found in both datasets and not to all glycosites or even more, the full protein sequence. This is because we find important, that unlike in typical proteomics comparisons, additional information should be considered like glycosite specific regulation, macro- and micro-heterogeneity or isoform-specific glycosite annotation. We also evaluated the combined dataset for identification of potential new substrates of class I mannosidases other than EDEM2. For this, we sub-selected glycosites with no relative differences between CTR and siEDEM2 treated cells, but which were found as up-regulated glycosites in the current dataset. In this category we found other members of the integrin family (Integrin alpha-2 – ITGA2, Integrin alpha-5 – ITGAV), or lysosomal proteins involved in cholesterol export (SCARB2) (Heybrock et al., 2019), each with two distinct glycosites, but also entries with a single N-glycosylation site like HLA class II histocompatibility antigen, DRB1 beta chain (HLA-DRB1) or Transmembrane emp24 domain-containing protein 4 (TMED4, Figure 4D and Supplementary Table S3). These could represent candidates for other class I mannosidases and their degradation or maturation could rely on alternative routes, independently of EDEM2 selection. Further analysis of the core-fucosylated glycosites found in the EDEM2 dataset did not reveal a similar pattern as the one in kifunensine treated cells (Figure 4F), although we observed sites with negative log differences. In conclusion this comparison confirms our approach that indeed most of the kifunensine upregulated glycosites are from ESP, particularly proteins aimed for maturation and/or degradation, which further validates our findings using this workflow.

### 3.6 Western Blot analysis confirms the identification of class I mannosidase clients regardless of their intracellular processing status

We further interrogated the up-regulation mechanism behind a few of class I mannosidases candidate substrates using SDS-PAGE coupled with Western Blot detection. We selected two of these from which we found up-regulated glycosites in both datasets, PCHDGC3 (PCDH2) a protein involved in cell-adhesion and soluble tyrosinase (TYR) from which we detected multiple glycosites upregulated in siEDEM2 and KIF treated cells (Figure 4D).

**FIGURE 5:**
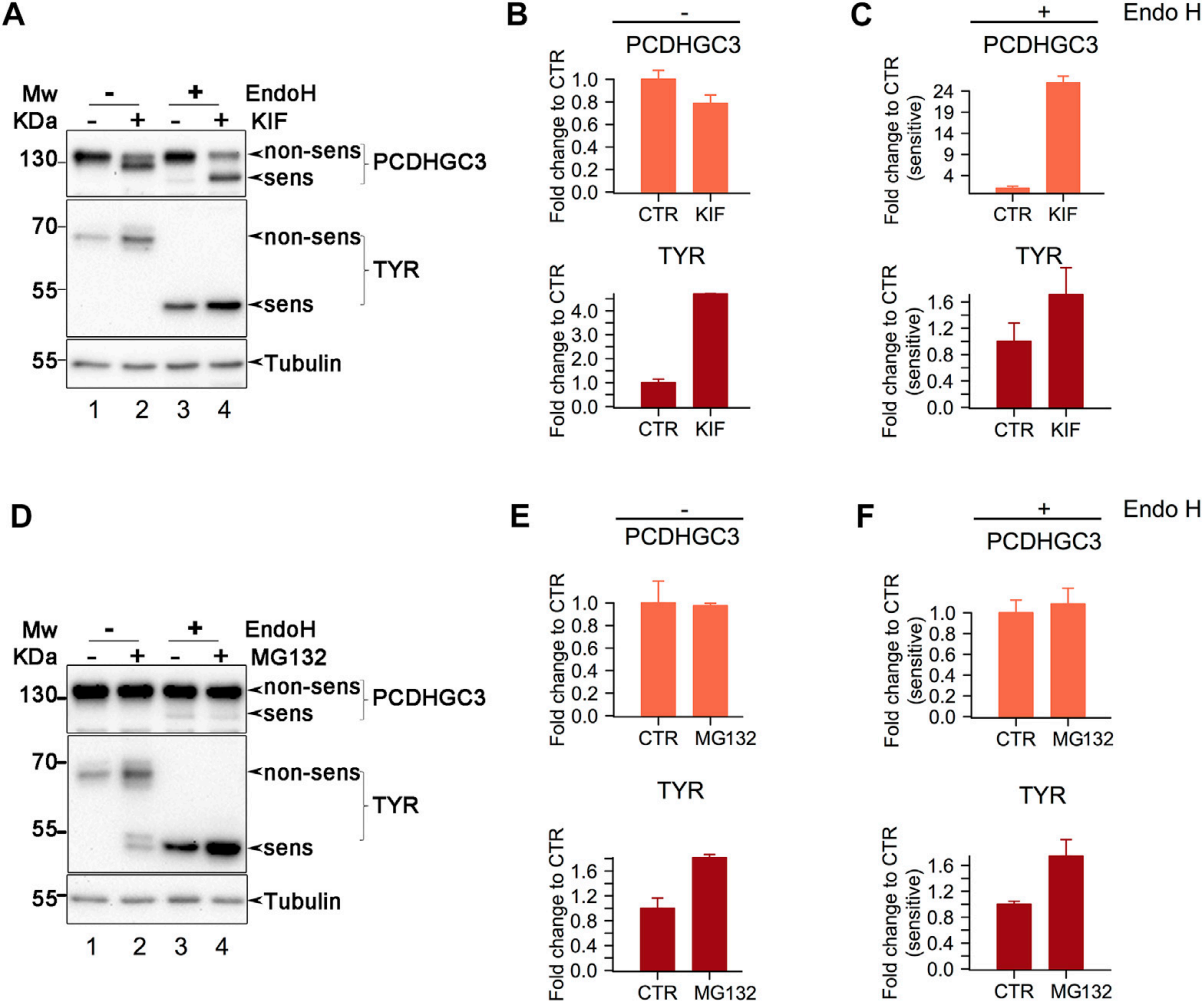
Western Blot analysis of class I mannosidase candidate substrates. **(A).** Analysis of cells treated with KIF. Cell lysates were split, and one part was subject to Endo H digestion. Proteins were then separated in polyacrylamide gels and transferred on nitrocellulose membrane which were further incubated with the designated antibodies. Shown is a representative experiment of duplicates samples. **(B, C).** Samples in A were subject to densitometry analysis and the obtained values were further used to assess the differences between the conditions. Bars are means of duplicate experiments and error bars are SEM. **(D).** Similar as in (A)., but the cells were treated with MG132, proteasome inhibitor. **(E, F).** Densitometry analysis of samples from (D). Bars are mean of triplicate experiments and error bars are SEM.

As can be observed in Figure 5A treatment of cells with kifunensine resulted in the appearance of multiple glycoforms not visible in CTR cells (Figure 5A, compare lanes 1 and 2), for both candidates PCDHGC3 (upper panel) and TYR (middle panel), suggesting class I dependent intracellular glycoprotein processing. Treatment with KIF also resulted in some alterations at the total protein level which were more pronounced for TYR (Figure 5B, compare upper and lower panels), revealing a net increase of the TYR relative levels and a slightly decrease for PCDHGC3. Thus, the overall proteins respond similarly regarding intracellular processing but different regarding their abundance, indicating that while for TYR, enzymes from class I mannosidases are required for its degradation, for PCDHGC3 these enzymes are required for glycoprotein maturation.

Endo H digestion resulted in the appearance of lower molecular weight products for both PCDHGC3 and TYR, indicating glycoform processing by the enzyme (Figure 5A, compare lanes 1 and 3 from upper and middle panels). Close analysis indicates that PCDHGC3 contains a minor proportion of molecules which are Endo H sensitive, suggesting that at steady-state, most of the molecules are mature glycoforms. In contrast TYR revealed that all the molecules are Endo H sensitive (Figure 5A, compare upper and middle panels for lanes 1 and 3), indicating that all the molecules have non-mature glycoforms, in agreement with previous findings (Popescu et al., 2005).

Both candidates revealed increased signal intensity for the Endo H products following KIF treatment of cells (Figure 5A, upper and middle panels, compare lanes 3 and 4). Comparison of the relative abundance of the Endo H fraction in KIF treated with CTR cells, revealed that there was a considerable increase of non-processed (immature) molecules following KIF treatment, for PCDHGC3, (Figure 5A upper panel, compare lanes 3 with 4, and Figure 5C upper panel). TYR displayed similar changes compared with control (Figure 5C, lower panel), although to a lesser extent. These results indicate that the accumulation observed following KIF treatment of the Endo H fraction of TYR and PCDHGC3 are similarly impacted. However there is a different response of the PCDHGC3 mature glycoforms, suggesting the existence of two different molecular pools for this protein, compared with TYR, which respond different to KIF treatment. The upregulation observed in Figure 5C, upper panel, could result following intracellular blockage of class I-dependent maturation events of PCDHGC3, or following to the blockage of degradation for the non-mature PCDHGC3 molecular pool.

To obtain further details about these aspects, we performed a similar experiment, but in which the cells were treated with MG132, an inhibitor of the proteasome which usually results in accumulation of the substrates which are sent to degradation towards the canonical ubiquitin-proteasome pathway. When cells were treated with MG132 we observed a net increase in the TYR level compared with the control, but no major difference for PCDHGC3, (Figure 5D, compare lanes 1 and 2 in upper and middle panels and Figure 5E, compare upper and lower panels), thus suggesting that the overall molecular pool of TYR and PCDHGC3 respond differently to proteasome inhibition.

Moreover, analysis of the Endo H digested samples revealed a similar pattern for TYR, as the one observed in the KIF treatment (Figures 5C, F, compare lower panels), indicating protein up-regulation. Analysis of PCDHGC3 suggested an upregulation of the Endo H sensitive fraction only (Figure 5F, upper panel). However, the difference was not large and this would be expected considering the minor fraction of Endo H sensitive PCDHCG3 molecules observed initially (Figures 5A, D, upper panels, lane 3). This in contrast with the total pool of PCDHGC3 molecules (Figure 5E, upper panel), which did not indicate a similar pattern. These results suggest that the increase up-regulation in KIF treated cells is a consequence of two events for PCDHGC3: a net overall blockage of the class I dependent maturation events but also due to the possible degradation blockage, to a lesser extent and only to the steady-state non-mature glycoforms molecular pool. However, we cannot exclude that PCDHGC3 degradation could be also-dependent on alternative routes which involves the cooperation of class I mannosidases, thus resulting in similar observations. Taken together, these results validate our findings using gESP, but the mechanism behind up-/down-regulation in KIF treated cells could be glycosite- or protein-specific.

## 4 Discussion

In this work we developed a workflow to characterize the glycoprotein members of the early secretory pathway (ESP) by integrating carbohydrate and partial deglycosylated peptide analysis using high-resolution mass spectrometry of kifunensine treated cells. Our workflow provides several advantages over the traditional glycoproteomics strategies: first using a combination of Concanavalin A affinity towards oligomannose containing structures and the Endo H specificity we can restrict the analysis only to the oligomannosidic glycoproteins as this is important since this fraction of molecules is expected to contain ESP glycoproteins at steady-state measurements inside the cell; second, it allows unambiguously N-glycosylation site localization by focusing the analysis only to HexNAc or dHexHexNAc containing glycopeptides; and third our workflow also reveals details about their associated carbohydrate structures and their maturation state. By analysing separately the glycopeptides and their glycan structures, we can obtain MS/MS spectra containing either peptide fragment ions or alternatively, carbohydrate fragmentation ions. This simplifies the computational analysis which is still resource-consuming comparing with the analysis of MS/MS spectra obtained from intact glycopeptide fragmentation. Still, this workflow provides a net advantage over the traditional PNGse-F deglycoproteomics approaches since the MS analysis is dedicated to real simplified versions of glycopeptides and not to regular deamidated peptides, which comes with several disadvantages in terms of glycosite localization (Palmisano et al., 2012). While the direct link between glycosite and N-glycan structure is lost, we consider that this approach, based on a combination of lectin affinity and Endo H specificity, combined with kifunensine treatment, provides the best outcome in the selection of ESP members, compared with more general approaches for glycopeptide enrichment like HILIC (Alpert, 1990). Although the Concanavalin A specificity is limited, we note that the additional step of Endo H digestion increases the enrichment efficiency focusing the analysis to HexNAc containing peptides. It is also possible that folded glycoproteins that pass Golgi and reach plasma membrane or are secreted can have oligomannosidic glycosites (Hall et al., 2013). However, the results obtained following siEDEM2 dataset comparison do not sustain this hypothesis. If we combine this with the observation that only a small fraction can reach plasma membrane as oligomannose-type (Hall et al., 2013) and furthermore, that a great proportion of the identified glycosites are from ER-transitory proteins we conclude that at least a fraction of the identified glycosites can be attributed to the non-matured glycoproteins. Furthermore, in the context of N-linked glycan trimming inhibition, our approach provides a view also on the main pillars of the ESP glycoproteins which are subject to class I mannosidases recognition and degradation. Here we find that most of these are members of the glycocalyx, typically involved in cell adhesion and cell communication and that glycoproteins associated with terms such as proteolysis or protein folding are negatively associated with kifunensine treatment (Figure 2H). It is interesting to note that kifunensine does not impact all glycoproteins in the same way. For instance, some glycoproteins such as BSG or LAMC1 revealed similar regulation across the identified glycosites suggesting protein-level regulation, while others such as ECE1 or PSAP displayed considerable variation across the identified glycosites. This suggests that additional factors contribute to the site-level regulation beside the steady-state protein level, such as macro- and micro-heterogeneity factors. This cannot be explained by the variation in instrument analysis or sample preparation as these would impact in a similar manner all the quantified glycosites and not particular glycosites.

At the glycan level we observed a net increase of the relative abundance for G0M9 structures, implying that most of the glycosites found as up-regulated in kifunensine treated cells compared with CTR were most probably carrying this carbohydrate structure. A similar observation was reported for triple knockout (KO) cells for two Golgi mannosidases (MAN1A1, MAN1A2) and the ER-located MAN1B1 (Jin et al., 2018). An unexpected result of kifunensine treatment is related towards the relative abundance of dHexHexNAc modified peptides (ones carrying FucGlcNAc), which were found, in general, with negative log ratios when comparing KIF treated with CTR cells (Figure 3B). This observation is even more evident when comparing the ratios of the corresponding HexNAc glycopeptides from the same glycoproteins which did not reveal similar differences (Figure 3C), thus suggesting that this is a site-directed effect and not a protein level-regulated one. Moreover, this result is partially explained by the lower expression of FUT8 in kifunensine treated cells, (Figure 3D), although we cannot totally rule out that this could also involve a reduction in its enzymatic activity. From our knowledge this is the first report providing glycosite level resolution for such effect on endogenous glycoproteins induced by kifunensine treatment; so far this has been reported only for recombinant glycoprotein overexpression systems (Zhou et al., 2008). This is important since core-fucosylation is also involved in immune recognition (Fujii et al., 2016) and also has been recently linked to melanoma metastasis (Agrawal et al., 2017). Moreover FUT8 upregulation also has been described in various cancer pathologies like hepatocellular carcinoma, pancreatic, breast, lung and colorectal cancer or in epithelial-mesenchymal transition (Chen et al., 2013; Liao et al., 2021). There are several mechanisms which could results in FUT8 upregulation in cancer among them implying microRNAs. More specifically the level of FUT8 expression was correlated with miR-10 b co-upregulation *via* the transcription factor Twist or the transcription factor activator protein 2γ (AP-2γ) in breast cancer cells (Guo et al., 2018; Ma et al., 2021). Other studies have indicated inversely correlated levels of miR-26a, miR-34a, and miR-455–3p with FUT8 mRNA, by analysis of 27 hepatocellular carcinoma tissue samples using qRT-PCR (Bernardi et al., 2013; Cheng et al., 2016). We also compared our results with a previous dataset in which one of the key-mannosidases involved in misfolded glycoprotein recognition (EDEM2) was downregulated using siRNA targeting the endogenous EDEM2 and validated our previous EDEM2 candidate-substrates such as ITGA1, PCDH2, HLA-B or tyrosinase, emphasizing the merits of our approach in the identification of bona fide endogenous candidate substrates from melanoma cells. These are important since from this pool of molecules, HLA-associated peptides are presented on the cell surface for immune recognition by cytotoxic CD8^+^ T-cell. Moreover, one aspect of this process is that the endogenous level of HLA is subject to the same pathway of gpERAD regulation, thus suggesting a mechanism by which cancer cells can become ‘invisible’ to the cell-specific immune surveillance. Moreover, this is also reinforced by the recent findings of our group regarding the key-role of EDEM proteins in protecting organisms in response to stress factors (Chiritoiu et al., 2020; Ghenea et al., 2022). Additionally, close comparison of kifunensine and EDEM2 datasets suggest that downregulation of core-fucosylation could be a kifunensine-specific consequence (Figure 4F). An interesting question arises regarding the mechanism involved behind glycosite upregulation in both, KIF and siEDEM2 treated cells. As mentioned earlier this could be a consequence of glycoform maturation events blockage or due to class I mannosidase-dependent degradation blockage, or due to both. However, these could also imply EDEM2 cooperation which, until now, was shown to function only as a class I-like mannosidase in misfolded protein proteasomal degradation. Alternative degradation routes like ERLAD or ER-phagy cannot be excluded (Fregno et al., 2018; Chiritoiu et al., 2020; Fregno et al., 2021). Further experiments could elucidate the molecular mechanisms observed behind glycosite up/down-regulation in KIF treated cells.

## Data Availability

The raw data associated with this article have been deposited to the ProteomeXchange Consortium via the PRIDE (Perez-Riverol et al., 2019; Deutsch et al., 2020) partner repository with the dataset identifier PXD035541.
