# Supplementary material for: Defining the altered glycoproteomic space of the early secretory pathway by class I mannosidase pharmacological inhibition
Source: Front Mol Biosci. 2023 Jan 9;9:1064868. doi: 10.3389/fmolb.2022.1064868 (PMC9869281; doi:10.3389/fmolb.2022.1064868)
Supplement: Supplementary file 1 [file DataSheet1.pdf]

Supplementary Material

1 Supplementary Figures

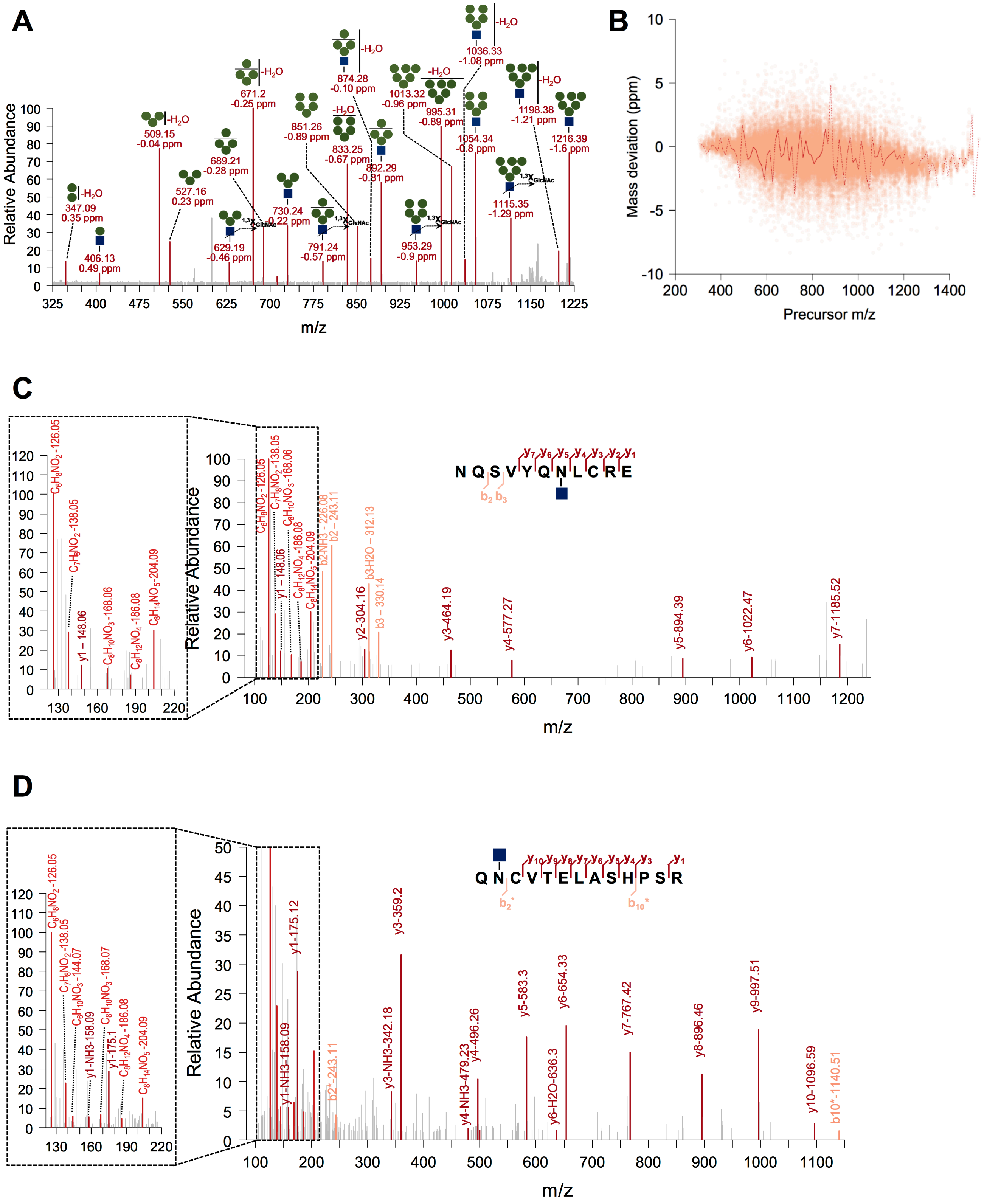
A
B
C
D
Relative Abundance
m/z
347.09
0.35 ppm
406.13
0.49 ppm
509.15
-0.04 ppm
527.16
0.23 ppm
629.19
-0.46 ppm
671.2
-0.25 ppm
689.21
-0.28 ppm
730.24
-0.22 ppm
791.24
-0.57 ppm
833.25
-0.67 ppm
851.26
-0.89 ppm
874.28
-0.10 ppm
892.29
-0.81 ppm
953.29
-0.9 ppm
995.31
-0.89 ppm
1013.32
-0.96 ppm
1036.33
-1.08 ppm
1054.34
-0.8 ppm
1115.35
-1.29 ppm
1198.38
-1.21 ppm
1216.39
-1.6 ppm
-H2O
Mass deviation (ppm)
Precursor m/z
NQSVYQNLCRE
QNCVTELASHPSR
C6H8NO2-126.05
C7H8NO2-138.05
C8H10NO3-168.06
C8H12NO4-186.08
C8H14NO5-204.09
y1-148.06
b2-NH3-226.08
b2-243.11
b3-H2O-312.13
b3-330.14
y2-304.16
y3-464.19
y4-577.27
y5-894.39
y6-1022.47
y7-1185.52
C6H10NO3-144.07
C8H10NO3-168.07
y1-NH3-158.09
y1-175.1
y1-175.12
b2*-243.11
y3-NH3-342.18
y3-359.2
y4-NH3-479.23
y4-496.26
y5-583.3
y6-H2O-636.3
y6-654.33
y7-767.42
y8-896.46
y9-997.51
y10-1096.59
b10*-1140.51

Supplementary Figure 1. Analysis of gESP identified carbohydrate structures and their corresponding glycopeptides. (A). High-resolution MS/MS detection of the fragments corresponding to G0M6 fragmentation. Sown are the m/z, mass accuracy and the assigned structures. All ions were detected as +1 sodium adducts. (**B**)**.** Mass deviation of precursors corresponding to all of the identified glycopeptides. As can be observed most of these are well below sub-ppm range. (**C**)**.** High-resolution HCD MS/MS fragmentation of the peptide NQSVYQN(HexNAc)LCRE obtained following GluC digestion. It can be observed the identification of y5-y7 ions demonstrating the position of the HexNAc relative to the two Asn residues, together with b2 and b3 ions. (**D**)**.** Similar as in C, but for the glycopeptide QN(HexNAc)CVTELASHPSR. * denotes ions which lost the HexNAc residue during fragmentation.

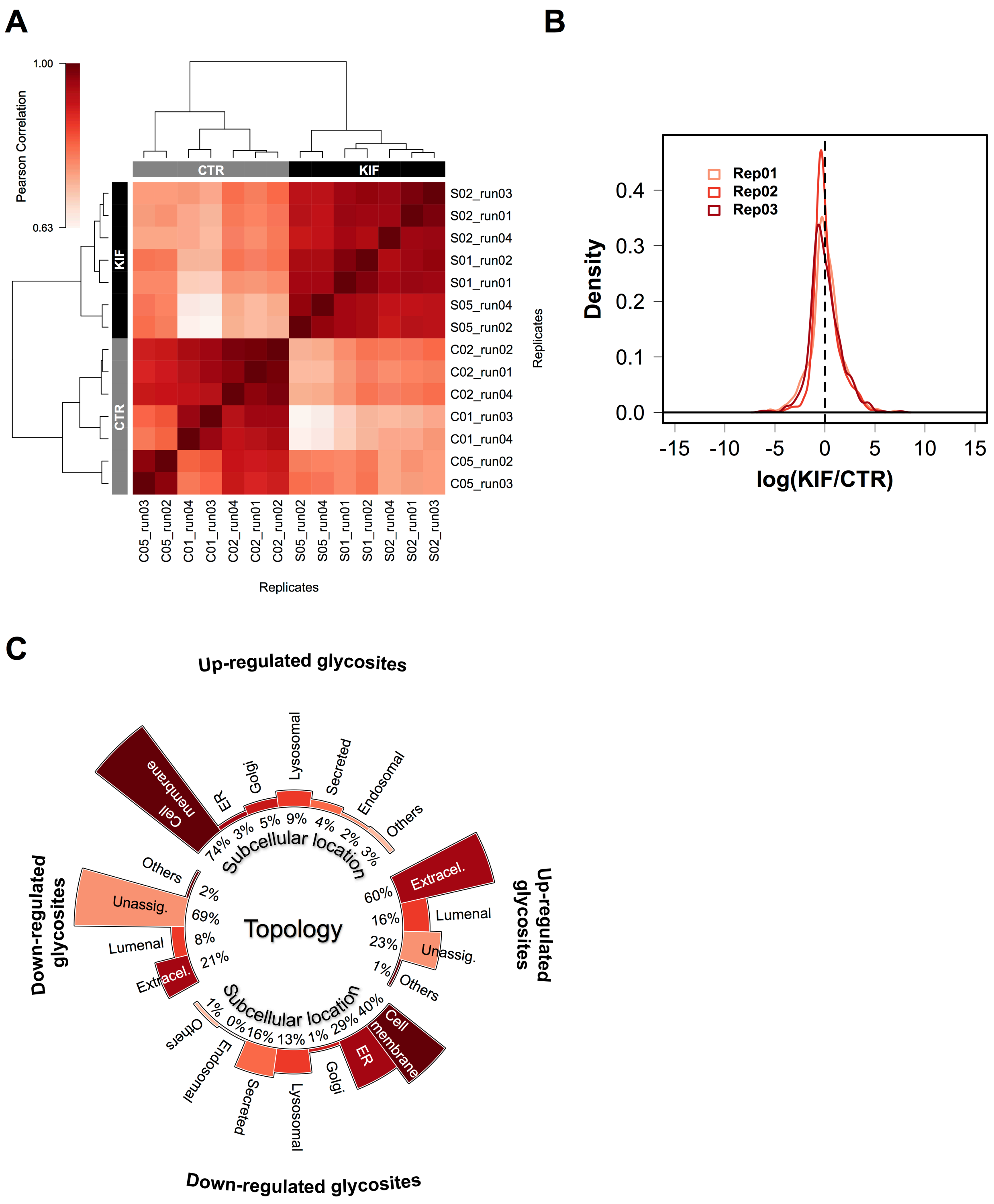
A
B
C
1.00
0.63
Pearson Correlation
CTR
KIF
S02_run03
S02_run01
S02_run04
S01_run02
S01_run01
S05_run04
S05_run02
C02_run02
C02_run01
C02_run04
C01_run03
C01_run04
C05_run02
C05_run03
C05_run03
C05_run02
C01_run04
C01_run03
C02_run04
C02_run01
C02_run02
S05_run02
S05_run04
S01_run01
S01_run02
S02_run04
S02_run01
S02_run03
Replicates
Replicates
Rep01
Rep02
Rep03
Density
0.4
0.3
0.2
0.1
0.0
-15
-10
-5
0
5
10
15
log(KIF/CTR)
Up-regulated glycosites
Cell membrane
ER
Golgi
Lysosomal
Secreted
Endosomal
Others
74%
3%
5%
9%
4%
2%
3%
Subcellular location
Topology
Extracel.
Lumenal
Unassig.
Others
60%
16%
23%
1%
Up-regulated glycosites
Down-regulated glycosites
Others
Unassig.
Lumenal
Extracel.
2%
69%
8%
21%
Subcellular location
1%
0%
16%
13%
1%
29%
40%
Cell membrane
ER
Golgi
Lysosomal
Secreted
Endosomal
Others
Down-regulated glycosites

Supplementary Figure 2. Quantitative glycoproteomic analysis of KIF treated cells. (A). Heatmap of Pearson correlation coefficients between technical and biological replicates. It can be observed the specific clustering of CTR and KIF treated cells, which demonstrate clear discrimination at the glycopeptide level between the two conditions. (**B).** Distribution analysis of the log fold changes for the analyzed biological replicates. The calculated ratios show an almost zero-centered normal distribution. (**C).** Topological and subcellular localization of the differential regulated glycosites in KIF treated cells. For protein topological annotation, information from the UniProtKB database was used.
